# The possible role of visceral fat in early pregnancy as a predictor of gestational diabetes mellitus by regulating adipose-derived exosomes miRNA-148 family: protocol for a nested case-control study in a cohort study

**DOI:** 10.1186/s12884-021-03737-1

**Published:** 2021-03-30

**Authors:** Zhenhong Zhang, Qian Xu, Yanping Chen, Lun Sui, Lu Jiang, Qianqian Shen, Minyu Li, Guoju Li, Qiuzhen Wang

**Affiliations:** 1 Public Health School, Medical College of Qingdao University, Qingdao, China; 2 Qingdao Women and Children’s Hospital, Qingdao University, No.6 Tongfu Road, Qingdao, 266000 Shandong Province China

**Keywords:** Visceral fat, Adipose-derived exosomes, miRAN-148 family, Gestational diabetes mellitus

## Abstract

**Background:**

Gestational diabetes mellitus (GDM) has become alarming public health concern. It is associated with adverse pregnancy outcomes and increased risk of postpartum type 2 diabetes. Pre-pregnant body mass index (BMI), waist circumference and other anthropometric parameters have been proposed to predict GDM. However, visceral fat thickness can better reflect the distribution of body fat, and may more accurately predict the risk of GDM. Visceral fat thickness may lead to insulin resistance by regulating the adipose-derived exosomes miRNA-148 family, which affect the development of GDM. Evidence from prospective cohort studies on visceral fat thickness as a predictor of GDM and the possible mechanisms is still insufficient.

**Methods:**

In this prospective cohort study, we will recruit 3000 women at first antenatal visit between 4 and 12 weeks of gestation. Baseline socio-demographic factors and visceral fat thickness will be assessed by questionnaire form and the ultrasonic measurement, respectively. At 20 weeks of gestation, 10 ml blood samples will be drawn and we will extract adipose-derived exosomes miRNA on the basis of nested case-control study. GDM will be screened at 24–28 weeks’ gestation and the expression of miRNA-148 family between pregnant women with GDM and without GDM will be analyzed. Intermediary analysis will be used to investigate whether visceral fat thickness can predict GDM by regulating adipose-derived exosomes miRNA-148 family.

**Discussion:**

We hypothesized that visceral fat thickness may predict GDM by regulating the miRNA-148 family of adipose-derived exosomes. The findings of the study will assist in further clarifying the pathophysiological mechanism of GDM, it will also provide technical support for effective screening of high-risk pregnant women with GDM.

**Supplementary Information:**

The online version contains supplementary material available at 10.1186/s12884-021-03737-1.

## Background

Gestational diabetes mellitus (GDM) is a common complication of pregnancy, affecting 7–25% of pregnancies worldwide [1, 2], forming a severe public health concern. GDM contributes to the occurrence of preeclampsia [3], gestational hypertension [4], macrosomia [5] and preterm birth [6]. It is also associated with long-term adverse outcomes in postpartum women and offspring, including increased risk of developing obesity [7], cardiovascular disease [8] and type 2 diabetes [9]. Previous study reported that the incidence of GDM in Asia was as high as 20.9% [10]. A systematic review [11] suggested that the incidence of GDM in Chinese mainland was 14.8%, which indicated that China might have the largest number of GDM patients in the world. The city of Qingdao, the cite of our field work, had an incidence of GDM as 17.4% [12], which was higher than that reported as the average level in the mainland of China. In recent years, there is an increasing trend in the prevalence of GDM globally, including in China [13–15]. In view of the adverse consequences and high prevalence of GDM, identifying the major risk factors for GDM in an early stage of the pregnant is essential.

Risk factors such as pre-pregnancy overweight and obesity [16], advanced maternal age [17], family history of diabetes [18] have been proposed to be related to the incidence of GDM, previously. However, different from those constant risk factors, overweight/obesity is a potentially modifiable factor of GDM. Accordingly, body mass index (BMI), waist circumference and other related anthroprometric parameters have been proposed to predict GDM. However, their predictive power for GDM was controversial. Visceral fat, better reflecting of the distribution of body fat [19], has been reported to play an important role in the prevalence of GDM [20, 21]. A cohort study [22] showed that visceral fat may be a powerful predictor of GDM. Additionally, another study concluded that the accuracy of visceral fat thickness in early pregnancy to predict GDM may be better than that of BMI [20]. However, the pathogenesis of GDM due to visceral fat remains largely unknown. In recent years, studies have shown that adipose-derived exosomes play a significant role in the occurrence and development of GDM. The research results published on journal of Cell [23] in 2017 showed that adipose tissue in obese mice secreted miRNA-containing exosomes, which induced glucose intolerance and insulin resistance when administered to lean mice. In the process, adipose-derived exosomes miRNA played an important role. Another research indicated that visceral adipose-derived exosomes participated in receptor-insulin signal transduction through down-regulation of miRNA-148-a and miRNA-148-b expression [24–26]. Therefore, the abnormal expression of miRNA in adipose-derived exosomes caused by visceral fat accumulation may be an important mechanism in the pathogenesis of GDM.

We hypothesized that visceral fat thickness may predict GDM by regulating the adipose-derived exosomes miRNA-148 family, and verify this hypothesis in a cohort & nested case-control study.

## Methods/design

### Study design

This is a prospective cohort study, and it will follow the requirements of strengthening observational study reports in the guidelines for epidemiological reporting of cohort studies. Meanwhile, in order to explore the mechanism of visceral fat thickness influencing GDM by regulating adipose-derived exosomes miRNA, we organized a 1:1 nested case-control study according to age ± 2 years old from the prospective cohort of pregnant women.

### Study objectives

1. To explore the influence of BMI and visceral fat thickness in early pregnancy on GDM, and select the appropriate adiposity indicator.
2. To evaluate the expression of adipose-derived exosomes miRNA-148 family in pregnant women with GDM and without GDM, and study how visceral fat thickness regulates the miRNA-148 family of adipose-derived exosomes to cause GDM.

### Recruitment of participants

Study recruitment started on 1 April 2019 at Qingdao Women and Children’s Hospital and it is expected to last until April 2021. It will have to meet the following criteria:

#### Inclusion criteria

(1) Singleton pregnancy; (2) First antenatal visit is 4–12 weeks of gestation; (3) Age ≥ 20 years; (4) The target pregnant women are permanent residents in Qingdao.

#### Exclusion criteria

(1) Without definite date of last menstruation; (2) With kidney and other endocrine diseases; (3) With cardiovascular and cerebrovascular diseases and important organ dysfunction; (4) Refusal of follow-up.

### Plan of follow-up

**Fig. 1:**
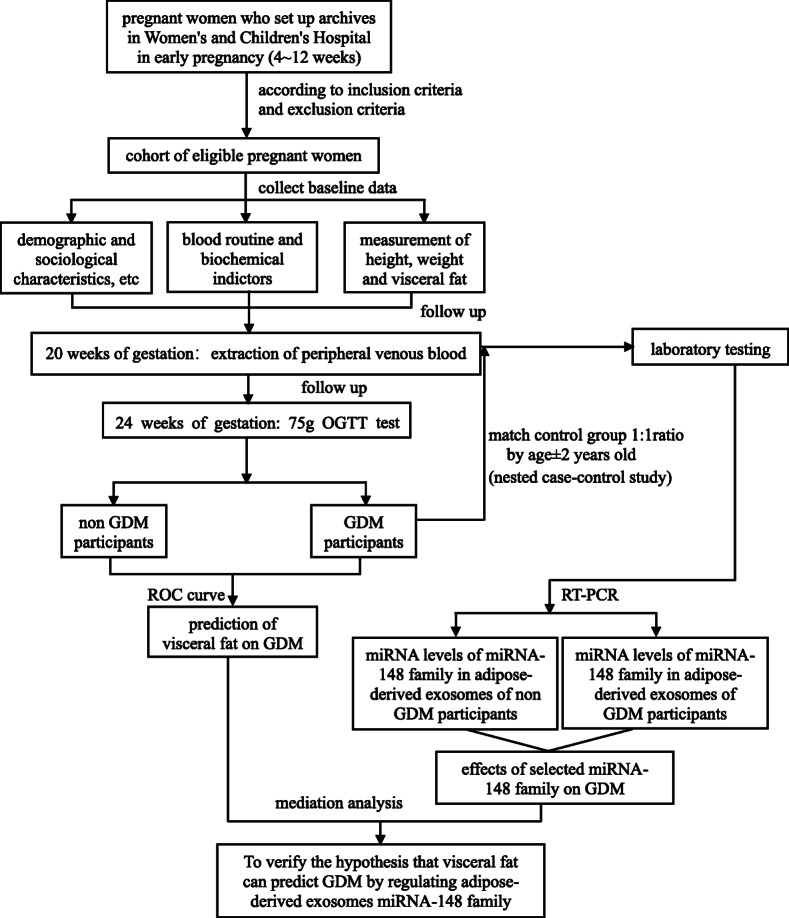
Technical roadmap of this research project Note: inclusion criteria: singleton pregnancy, first antenatal visit is 4–12 weeks of gestation and aged 20 years and above. Exclusion criteria: multiple pregnancy, no definite time of last menstruation, have kidney and other endocrine diseases, combined with cardiovascular and cerebrovascular diseases and important organ dysfunction and those who can’t complete the follow-up. control group: non-GDM participants

**Fig. 2:**
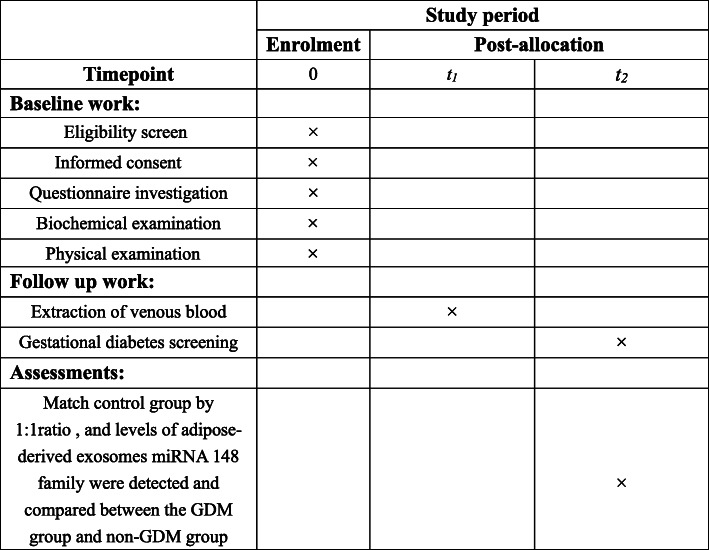
Schedule of enrolment, follow-up and assessments

At 20 weeks of gestation, 10 ml blood samples will be drawn and we will extract adipose-derived exosomes miRNA. GDM will be screened at 24–28 weeks’ gestation and we organized a 1:1 nested case-control study according to age ± 2 years old from pregnant women, the expression of miRNA-148 family between pregnant women with GDM and without GDM will be analyzed. Technical roadmap of this research project can be found in Fig. 1 and a schematic schedule of enrolment and assessments can be found in Fig. 2.

### Outcome measures

#### Primary outcomes

The central outcome variable is GDM. During 24–28 weeks of gestation, 75 g oral glucose tolerance test (OGTT) will be carried out to detect the blood glucose level. According to the International Association of Diabetes and Pregnancy Study Groups (IADPSG) standard [27], GDM will be diagnosed if subjects meet at least one of the following criteria: (1) fasting venous blood glucose of ≥5.1 mmol/L; (2) 1-h venous blood glucose of ≥10.0 mmol/L; (3) 2-h venous blood glucose of ≥8.5 mmol/L.

#### Secondary outcomes

The secondary outcomes of this study include: weight gain and results of blood pressure monitoring at 20 and 24–28 weeks of gestation, respectively.

### Data collection

#### Research questionnaire form

Sociodemographic information include age, education level, personal monthly income and working status during pregnancy will be collected by questionnaire. Behavior and lifestyle include smoking, drinking and physical activity. Parity, personal and family disease history will also be collected. The questionnaire is shown in Additional file 1.

#### Physical measurement

Standardized measurements of height, weight, waist circumference will be performed, and blood pressure will also be measured by using an internationally certified Omron electronic sphygmomanometer (hem-7220). In addition, visceral fat thickness will be measured by ultrasonographic, defined as the distance between the internal face of reto-abdominal muscle and the anterior wall of the aorta [28]. The standardized measurement method greatly reduces the potential data deviation and ensures the real reliability of the data.

#### Laboratory measurement

In the Clinical Laboratory of Qingdao Women’s and Children’s Hospital, routine blood, blood biochemical indices, and adipose-derived exosomes will be carried out. Based on the experimental method of Real-Time Fluorescence Quantitative PCR (RT-PCR), the miRNA-148 family of adipose-derived exosomes will be sequenced between pregnant woman with GDM and without GDM.

### Sample size calculation

In this study, obesity was selected as the main exposure indictor to calculate the sample size, and sample size has been calculated to reach a confidence level of 95% with a power of 80% and α significance level of 0.05. According to the following formula:

$$ \mathrm{N}={\left({\mathrm{z}}_{\upalpha}\sqrt{2\overline{\mathrm{p}\mathrm{q}}}+{\mathrm{z}}_{\upbeta}\sqrt{{\mathrm{p}}_0{\mathrm{q}}_0+{\mathrm{p}}_1{\mathrm{q}}_1}\right)}^2/{\left({\mathrm{p}}_1-{\mathrm{p}}_0\right)}^2 $$

In the light of the incidence of GDM in pregnant women with obesity before pregnancy was p1 =0.1378, q1 =0.8622 and the control group was p0 = 0.0564, q0 = 0.9436, $\overline{\mathrm{p}}$ =0.0971, $\overline{\mathrm{q}}$ =0.9029. If we assume a dropout rate of 10%, taking into account the unqualified samples and expanding sample proportion of cluster sampling to 1.7 times, the sample size of each group was 600. In order to fully analyze the risk factors of GDM, lay the foundation for the follow-up cohort, and finally determine each group of 1500 people.

Based on the above information, a nested case-control study was designed to analyze the miRNA-148 family expression between pregnant women with GDM and without GDM. Briefly, we will select all GDM subjects among members of the cohort and match them with control subjects who reach the age ± 2 years at the time in a 1:1 ratio.

### Ethics and dissemination

The study was approved by the Ethics Committee of Qingdao Women’s and Children’s Hospital (Number: 019–2019-FEKY). Each adverse event will be reported in writing to the Ethics Committee and each revision of the study protocol will also be reported. Investigators will strictly abide by the stated commandments of Helsinki and the ethical issues of human biomedical research when conducting this study. The patient has given informed consent to participate in the study, and patient information will remain confidential throughout the study process.

### Statistical analysis

Statistical analyses will be performed by using SAS 9.4 and the level of significance will be set at *p* < 0.05. First, the continuous variables will be tested for normality and homogeneity of variance, If the data conform the normal distribution, participant characteristics will be described mean ± standard deviation, and the comparison between the two groups will be used independent sample t-test; If the data does not conform the normal distribution, participant characteristics will be described interquartile ranges, and nonparametric test will be used to compare the two groups. For qualitative data, participant characteristics will be percentage, and chi square test will be used for comparison between groups. Covariate adjusted logistic regression will be used to analyze the possible influencing factors of GDM. Receiver Operator Characteristic (ROC) curve will be used to identify the best cut-off value of visceral fat thickness and BMI to predict GDM among all patients. The area under the ROC curve (AUC) of visceral fat thickness and BMI will be compared by z-test and we will select the appropriate adiposity indicator. Multiple imputation will be used for missing data.

**Fig. 3:**
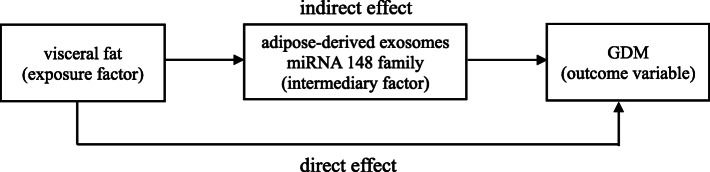
Simple sketch map of mediation

Mediation analysis will be used to explore the relationship between visceral fat thickness and GDM by regulating adipose-derived exosomes miRNA-148 family. It divides the effect of an exposure on the outcome to two parts, one is the direct effect and the other is the indirect effect. The effect of the visceral fat (exposure factor) on the GDM that is not through the adipose-derived exosomes miRNA-148 family is referred to as a direct effect. The effect of the visceral fat (exposure factor) on the GDM that operates through adipose-derived exosomes miRNA-148 family is referred to as an indirect effect. And the adipose-derived exosomes miRNA-148 family is referred to as “mediator”. The sketch map was shown in Fig. 3.

### Study status

Recruitment has started April 2019 and it is estimated it will take 3 years for full recruitment.

## Discussion

Our study is a population-based study aimed to assess accuracy of visceral fat thickness in predicting GDM and the visceral fat how regulates the miRNA-148 family of adipose-derived exosomes to cause GDM. Currently, more and more scholars discuss the predictive efficiency of anthropometric parameters for GDM [29–31]. However, there were few studies on the prediction of visceral fat thickness to GDM, moreover, the pathogenesis of visceral fat leading to GDM was unclear. At present, extensive efforts are being focused on the adipose-derived exosomes in relation to GDM. Previous study found that adipose-derived exosomes were the main source of circulating miRNA [32], these circulating miRNAs played an important role in regulating mRNA expression and translation. Adipose-derived exosomes miRNA were also associated with development of insulin resistance. The analysis of adipose-derived exosomes miRNA content pre- and post-gastric bypass showed upregulation of miR103-3p which was known to target the insulin receptor signaling pathway and was previously found to be downregulated in diabetes [33–35]. These studies demonstrated that adipose-derived exosomes can mediate gene regulation and functioning in distant cells. Therefore, in obese pregnancies, adipose-derived exosomes may communicate with the placenta and induce changes in its function which may contribute to the development of GDM. Thus, it is possible that adipose-derived exosomes were factors in the pathogenesis of GDM. However, there were few studies about visceral fat regulating the miRNA-148 family of adipose-derived exosomes to cause GDM, so we designed this study.

Our research has some advantages. Firstly, our case control study will be nested within a prospective cohort and the measurement of visceral fat thickness and adipose-derived exosomes miRNA-148 family will prior to the diagnosis of GDM, which allow us to estimate the causal relationship of visceral fat thickness on the development of GDM, although there are more and more studies on the effect of adipose-derived exosomes on diseases, our study nested case-control study on the basis of prospective cohort study, which is more scientific and reliable. Secondly, nested case-control study will save time, effort, and money. Thirdly, the GDM cases will be confirmed by medical records following a clear definition based on IADPSG guidelines rather than self-reported and it will guarantee reliability of results. However, on the basis of cohort study, a limitation of this study is loss of follow-up, but we will keep in touch with pregnant women at any time through wechat, which will greatly reduce our loss rate.

In conclusion, to a certain extent, the study is helpful to verify the effect of visceral fat on the development of GDM, and it is helpful to further clarify the pathophysiological mechanism of gestational diabetes. It also provides technical support for effective screening of high-risk pregnant women with GDM.

## Supplementary Information

**Additional file 1.** Questionnaire.

## Data Availability

The anonymised datasets used and/or analysed during the current study will be available from the corresponding author on reasonable request.

## Abbreviations

GDM: Gestational diabetes mellitus
BMI: Body mass index
OGTT: Oral glucose tolerance test
IADPSG: International Association of Diabetes and Pregnancy Study Groups
RT-PCR: Real-Time Fluorescence Quantitative PCR
ROC: Receiver Operator Characteristic
