# Correction to: The possible role of visceral fat in early pregnancy as a predictor of gestational diabetes mellitus by regulating adipose-derived exosomes miRNA-148 family: protocol for a nested case-control study in a cohort study

**DOI:** 10.1186/s12884-021-04109-5

**Published:** 2021-09-24

**Authors:** Zhenhong Zhang, Qian Xu, Yanping Chen, Lun Sui, Lu Jiang, Qianqian Shen, Minyu Li, Guoju Li, Qiuzhen Wang

**Affiliations:** 1 Public Health School, Medical College of Qingdao University, Qingdao, China; 2 Qingdao Women and Children’s Hospital, Qingdao University, No.6 Tongfu Road Shandong Province, Qingdao, 266000 China

**Correction to: BMC Pregnancy Childbirth 21, 262 (2021)**

**https://doi.org/10.1186/s12884-021-03737-1**

Following publication of the original article [1], the authors reported an error in the author-group. Qiuzhen Wang is also the corresponding author.

Zhenhong Zhang^1†^, Qian Xu^2†^, Yanping Chen^2^, Lun Sui^2^, Lu Jiang^2^, Qianqian Shen^1^, Minyu Li^1^, Guoju Li^2*^ and Qiuzhen Wang^1*^

The original article has been updated.
