# Supplementary material for: The possible role of visceral fat in early pregnancy as a predictor of gestational diabetes mellitus by regulating adipose-derived exosomes miRNA-148 family: protocol for a nested case-control study in a cohort study
Source: BMC Pregnancy Childbirth. 2021 Mar 30;21:262. doi: 10.1186/s12884-021-03737-1 (PMC8011135; doi:10.1186/s12884-021-03737-1)
Supplement: Supplementary file 1 — Additional file 1. Questionnaire. [file 12884_2021_3737_MOESM1_ESM.pdf]

Number:

Health Survey of Pregnant Women in Early Pregnancy in Qingdao

**Informed consent**	The Health Survey and Physical Examination were funded by National Natural Science Foundation of China and Qingdao application project.<br>**Purpose and content**: To investigate the health status of pregnant women in Qingdao. And general information and physical examination will be obtained during the survey. The contents of physical examination include visceral fat measurement, subcutaneous fat measurement, biochemical test, etc.<br>**Possible risks**: The risk of this investigation will not be greater than that of routine medical examination.<br>**Benefits**: You will get a free physical examination report and expert advice that can reflect your health status.<br>**Confidentiality undertaking**: We guarantee that your personal information and inspection results will not be disclosed in any way under any circumstances.<br>**Voluntary principle**: This survey and physical examination are voluntary and you can refuse to participate or withdraw at any time.<br>**If you have suffered from severe diseases such as heart disease before, or have implanted artificial parts such as pacemaker in your body, please inform the registration personnel at the time of registration, and you will get our additional care during the physical examination.**<br>**I have had a detailed communication with the members of the research group to fully understand the purpose, content, possible risks and benefits of this survey. I voluntarily participate in this survey.**<br>**Signature of the respondents:______________**<br>**Date of signature:______________**

**personal information**	**Name**: _________<br>**ID number**: □□□□□□□□□□□□□□□□□□<br>**Actual date of birth（solar calendar）**: ____________<br>**Husband's actual date of birth（solar calendar）**: ____________<br>**Registered residence**: Qingdao City _________area；<br>□ Other places； **current residence**: Qingdao City _________area<br>**Current residence**: □ 1.town； □ 2. countryside<br>**Mobile number**: □□□□□□□□□□□ or □□□□□□□□□□□<br>**Is your menstruation regular:** □（1）It's basically regular （30±3day） □（2）Irregular<br>**Date of last menstruation（solar calendar）**: ____________<br>**What is the way of conception this time**: □ 1. nature conceived；<br>□ 2. in vitro fertilization、embryo transplantation；<br>□ 3. artificial insemination

Part one: Characteristics of population Sociology

1	**Level of education**: ☐ 1. illiteracy ☐ 2. primary school ☐ 3. junior middle school （technical school） ☐ 4. high school（polytechnic school） ☐ 5. undergraduate course （junior college） ☐ 6. Postgraduate and above
2	**Present occupation（You can choose more than one）**: ☐ 1.worker ☐ 2. peasant ☐ 3. Functionaries ☐ 4. salesperson ☐ 5. personnel in a specific technical field ☐ 6. student ☐ 7. housewife ☐ 8.Other_______
3	**Personal income**：monthly income ☐☐☐☐☐☐RMB **or** annual income ☐☐☐☐☐☐ RMB<br>**or** Household income ☐☐☐☐☐☐ RMB/year **and** family members ☐ number

Part two: History of gestation

4	**Previous history of pregnancy and childbirth**: **Apart from this pregnancy, have you ever been pregnant before** ☐**No** ☐**Yes**：number of pregnancy ☐ ☐，among them，**Abortion** (Number of spontaneous abortions ☐、 Number of induced abortion ☐)；**Live Birth**（Number of full-term live births ☐、Number of preterm births（Less than 37 gestational weeks） ☐）; **Number of stillbirths** ☐； **Number of neonatal deaths within 7 days** ☐.
5	**Is previous pregnancy pregnant with a defective child?（such as congenital heart disease、cheilopalatognathus、Down syndrome, etc.）**<br>☐No ☐Yes，Disease type _____________________<br>**Have you ever been bred fetal macrosomia?**（Weight ≥ 4000g within 1 hour after birth）<br>☐No ☐Yes

Part three: Status of disease

6	**Personal disease history**<br>Do you have diabetes ☐ 0.No ☐ 1.Yes ☐ 99. unclear<br>Type of diabetes： ☐ 1. Type 1 diabetes ☐ 2. Type 2 diabetes<br>Highest fasting blood glucose level: ☐☐. ☐ mmol/L<br>Highest postprandial blood glucose level: ☐☐. ☐ mmol/L<br>Date of diagnosis： _____________________<br>Diagnostic hospital： ☐ 1. Provincial Hospital ☐ 2. Municipal (District) hospital ☐ 3. county hospital ☐ 4.Other____ (village clinic, etc.)<br>therapeutic measure（**You can choose more than one**）:<br>☐ 1. Diet control ☐ 2. sports ☐ 3. Take hypoglycemic drugs ☐ 4. Insulin injection ☐ 5. Taking traditional Chinese Medicine ☐ 6. No treatment<br>Did you have gestational diabetes in your previous pregnancy？ ☐ 0.No ☐ 1.Yes ☐ 99. unclear

	Do you have high blood pressure? ☐ 0.No ☐ 1.Yes ☐ 99.unclear<br>The highest level of hypertension： systolic pressure ☐☐☐ mmHg diastolic pressure ☐☐☐ mmHg<br>Date of diagnosis： ____________________<br>Diagnostic hospital： ☐ 1. Provincial Hospital ☐ 2. Municipal (District) hospital ☐ 3. county hospital ☐ 4.Other____ (village clinic, etc.)<br>Did you have gestational hypertension in your previous pregnancy? ☐ 0.No ☐ 1.Yes ☐ 99.unclear<br>Do you have any urogenital diseases? ☐ 0.No ☐ 1.Yes ☐ 99.unclear, Date of diagnosis:________<br>Do you have polycystic ovary syndrome? ☐ 0.No ☐ 1.Yes ☐ 99.unclear, Date of diagnosis:________<br>Do you have thyroid disease? ☐ 0.No ☐ 1.Yes ☐ 99. unclear, Date of diagnosis:________<br>**Do you have any other diseases? (Heart disease, cancer, tuberculosis, asthma, etc)**<br>1.______, Date of diagnosis:________ 2._____, Date of diagnosis:________<br>3.______ ,Date of diagnosis:________ 4._____, Date of diagnosis:________
**7**	**Family history of disease（You can choose more than one）**<br>Who has diabetes in the family? ☐ 0.No ☐ 1.Yes ☐ 99.unclear<br>☐1.paternal-grandfather ☐2.paternal-grandmother ☐3. maternal-grandfather ☐4. maternal-grandmother<br>☐5.father ☐6.mother ☐7. brothers and sisters ☐8. sons and daughters<br>Who has hypertension in the family? ☐ 0.No ☐1.Yes ☐ 99.unclear<br>☐1.paternal-grandfather ☐2.paternal-grandmother ☐3.maternal-grandfather ☐4. maternal-grandmother<br>☐5. father ☐6. .mother ☐7. brothers and sisters ☐8. sons and daughters<br>Who has gestational diabetes in the family? ☐ 0.No ☐ 1.Yes ☐ 99.unclear<br>☐1. paternal-grandmother ☐ 2. maternal-grandmother ☐3.mother ☐4.sisters<br>Who has gestational hypertension in the family? ☐ 0.No ☐ 1.Yes ☐ 99.unclear<br>☐1. paternal-grandmother ☐ 2. maternal-grandmother ☐3. mother ☐4. sisters<br>Who has given birth to macrosomia in the family? ☐ 0.No ☐ 1.Yes ☐ 99.unclear<br>☐1. paternal-grandmother ☐ 2. maternal-grandmother ☐3. mother ☐4. sisters

Part four: life style

8 Smoking

	**6 months before pregnancy**		**First 3 months of pregnancy**	
Do you smoke?	□No	□Yes (How many cigarettes a day? _____)	□No	□Yes (How many cigarettes a day? _____)
Does your husband smoke?	□No	□Yes (How many cigarettes a day? _____)	☐No	☐Yes (How many cigarettes a day? _____)

	Are you exposed to secondhand smoke?	□No	□Yes (How many cigarettes a day? _____)	□No	□Yes (How many cigarettes a day? _____)
9	**Drinking**				
		**6 months before pregnancy**		**First 3 months of pregnancy**	
	Do you drink alcohol?	□No	□Yes (Do you drink white spirit, beer or red wine? How often? How much do you drink at a time?)	□No	□Yes(Do you drink white spirit, beer or red wine? How often? How much do you drink at a time?)
	Does your husband drink alcohol?	□No	□Yes (Do you drink white spirit, beer or red wine? How often? How much do you drink at a time?)	□No	□Yes (Do you drink white spirit, beer or red wine? How often? How much do you drink at a time?)

How many meals do you have on average before pregnancy? ☐1. One meal a day ☐2. Two meals a day ☐3. Three meals a day ☐4. Four meals a day

How many meals do you have on average during pregnancy? ☐1. One meal a day ☐2. Two meals a day ☐3. Three meals a day ☐4. Four meals a day

How about your appetite before pregnancy? ☐1.Good ☐2.Just so so ☐3.Bad

How about your appetite during pregnancy? ☐1.Good ☐2. Just so so ☐3.Bad

Do you have a partial diet before pregnancy? ☐0.No ☐1. Eat less vegetables ☐2. Eat less meat

Do you have a partial diet during pregnancy? ☐0.No ☐1. Eat less vegetables ☐2. Eat less meat

Which flavor do you like before pregnancy?（You can choose more than one） ☐1. light ☐2. moderate ☐3. salty ☐4. sweet ☐5. spicy ☐6. sour

Which flavor do you like during pregnancy?（You can choose more than one）☐1. light ☐2. moderate ☐3. salty ☐4. sweet ☐5. spicy ☐6. sour

What kind of cooking do you usually make before pregnancy? ☐1. water cooking ☐2. steam ☐3. stir-fry ☐4. deep frying ☐5.other_______

What kind of cooking do you usually make during pregnancy? ☐1. water cooking ☐2. steam ☐3.stir-fry ☐4. deep frying ☐5.other______

What kind of oil do you usually eat before pregnancy? ☐1. peanut oil ☐2. soybean oil ☐3. rapeseed oil ☐4. olive oil ☐5. corn germ oil ☐6. blend oil ☐7.other_______

What kind of oil do you usually eat during pregnancy? ☐1. peanut oil ☐2. soybean oil ☐3. rapeseed oil ☐4. olive oil ☐5. corn germ oil ☐6. blend oil ☐7.other_______

What kind of drink do you eat most before pregnancy? ☐1. plain boiled water ☐2. green tea ☐3. black tea ☐4. cola ☐5. fruit juice ☐6. coffee ☐7.other_______

What kind of drink do you eat most during pregnancy? ☐1. plain boiled water ☐2. green tea ☐3. black tea ☐4.cola ☐5. fruit juice ☐6. coffee ☐7.other______

What is your staple food before pregnancy? ☐1. steamed bread or rice ☐2 coarse food grain ☐3. tubers food ☐4. the three are basically equal

What is your staple food during pregnancy? ☐1. steamed bread or rice ☐2. coarse food grain ☐3. tubers food ☐4. the three are basically equal

What kind of food do you usually eat before pregnancy?(Sort by 1-3，1 is the most common eaten food)

1.__ 2.__ 3.__

a) meat b) liver and kidney of animal c) seafood d)egg e)milk f)tofu g) vegetables, fruits h) mushroom i) sweet and soft drinks k) no special

What kind of food do you not often eat before pregnancy?(Sort by 1-3，1 is the least commonly eaten food）

1.__ 2.__ 3.__

a) meat b) liver and kidney of animal c) seafood d)egg e)milk f)tofu g) vegetables, fruits h) mushroom i) sweet and soft drinks k) no special

What kind of food do you usually eat during pregnancy?(Sort by 1-3，1 is the most common eaten food)

1.__ 2.__ 3.__

meat b) liver and kidney of animal c) seafood d)egg e)milk f)tofu g) vegetables, fruits h) mushroom i) sweet and soft drinks k) no special

What kind of food do you not often eat during pregnancy?(Sort by 1-3，1 is the least commonly eaten food）

1.__ 2.__ 3.__

a) meat b) liver and kidney of animal c) seafood d)egg e)milk f)tofu g) vegetables, fruits h) mushroom i) sweet and soft drinks k) no special

Please recall whether you have eaten the following foods in the past year and estimate the frequency and average consumption of these foods

Food names	Times of eating							Average consumption
	Don't eat	< 1 time / week	1-3 times / week	4-6 times / week	Once a day	2-3 times / day	≥ 4 times / day	
		Please select the appropriate number of cycles to fill in						
1 Fruits (apple\banana, etc)								g
2 Dark leafy vegetables (spinach \rape\tomatoes, etc)								g
3 Light vegetable (Chinese cabbage\turnip, etc）								g
4 Mushroom								g
5 Salted products (pickles, etc)								g
6 Freshwater fish (carp\grass carp, etc)								g
7 Marine fish (Spanish mackerel, etc)								g
8 seafood (shrimp, etc)								g
9 poultry (chicken, etc)								g
10 meat (pork\beef, etc)								g
11 processed meat (sausage, etc)								g
12 nuts (almond\walnut, etc)								g
13 legume food (tofu, etc)								g or ml
14 dairy products(milk\yogurt, etc)								ml
15 egg (egg roll, etc)								g
16 cereals (steamed bun\rice, etc)								g

17 tubers food (potato, etc)								g
18 sweet food (bread\cake, etc)								g
19 fried food (Fried chicken, etc)								ml
20 soft drink (cola, etc)								ml
21 coffee								ml
22 tea (black tea\green tea, etc)								ml
23 edible oil								ml
24 salt								g
25 daily drinking water								ml

Please recall whether you have eaten the following foods since your pregnancy (early pregnancy) and estimate the frequency and average consumption of these foods

Food names	Times of eating							Average consumption
	Don't eat	< 1 time / week	1-3 times / week	4-6 times / week	Once a day	2-3 times / day	≥ 4 times / day	
		Please select the appropriate number of cycles to fill in						
1 Fruits (apple\banana, etc)								g
2 Dark leafy vegetables (spinach \rape\tomatoes, etc)								g
3 Light vegetable (Chinese cabbage\turnip, etc）								g
4 Mushroom								g
5 Salted products (pickles, etc)								g
6 Freshwater fish (carp\grass carp, etc)								g
7 Marine fish (Spanish mackerel, etc)								g
8 seafood (shrimp, etc)								g
9 poultry (chicken, etc)								g
10 meat (pork\beef, etc)								g 或 ml
11 processed meat (sausage, etc)								ml
12 nuts (almond\walnut, etc)								g
13 legume food (tofu, etc)								g
14 dairy products(milk\yogurt, etc)								g
15 egg (egg roll, etc)								g
16 cereals (steamed bun\rice, etc)								ml
17 tubers food (potato, etc)								ml
18 sweet food (bread\cake, etc)								ml
19 fried food (Fried chicken, etc)								ml
20 soft drink (cola, etc)								g
21 coffee								ml
22 tea (black tea\green tea, etc)								ml

23 edible oil								ml
24 salt								g
25 daily drinking water								ml

Folic acid	☐No	☐ Oral administration 3 months before pregnancy ☐Oral administration during 3 months of pregnancy ☐ Oral administration 3 months before pregnancy to the first 3 months after pregnancy	brand______ duration（month）______<br>☐Once a day ☐2 times/day ☐3 times/day<br>☐Once a week ☐>3 times / week<br>Dosage per time________
Iron	☐No	☐ Oral administration 3 months before pregnancy ☐Oral administration during 3 months of pregnancy ☐ Oral administration 3 months before pregnancy to the first 3 months after pregnancy	brand______ duration（month）______<br>☐Once a day ☐2 times/day ☐3 times/day<br>☐Once a week ☐>3 times / week<br>Dosage per time________
Calcium	☐No	☐ Oral administration 3 months before pregnancy ☐Oral administration during 3 months of pregnancy ☐ Oral administration 3 months before pregnancy to the first 3 months after pregnancy	brand______ duration（month）______<br>☐Once a day ☐2 times/day ☐3 times/day<br>☐Once a week ☐>3 times / week<br>Dosage per time________
Calcium magnesium mixture	☐No	☐ Oral administration 3 months before pregnancy ☐Oral administration during 3 months of pregnancy ☐ Oral administration 3 months before pregnancy to the first 3 months after pregnancy	brand______ duration（month）______<br>☐Once a day ☐2 times/day ☐3 times/day<br>☐Once a week ☐>3 times / week<br>Dosage per time________
Compound vitamin	☐No	☐ Oral administration 3 months before pregnancy ☐Oral administration during 3 months of pregnancy ☐ Oral administration 3 months before pregnancy to the first 3 months after pregnancy	brand______ duration（month）______<br>☐Once a day ☐2 times/day ☐3 times/day<br>☐Once a week ☐>3 times / week<br>Dosage per time________
Albumen powder	☐No	☐ Oral administration 3 months before pregnancy ☐Oral administration during 3 months of pregnancy ☐ Oral administration 3 months before pregnancy to the first 3 months after pregnancy	brand______ duration（month）______<br>☐Once a day ☐2 times/day ☐3 times/day<br>☐Once a week ☐>3 times / week<br>Dosage per time________
Fish oil	☐No	☐ Oral administration 3 months before pregnancy ☐Oral administration during 3 months of pregnancy ☐ Oral administration 3 months before pregnancy to the first 3 months after pregnancy	brand______ duration（month）______<br>☐Once a day ☐2 times/day ☐3 times/day<br>☐Once a week ☐>3 times / week<br>Dosage per time________
Cod-liver oil	☐No	☐ Oral administration 3 months before pregnancy ☐Oral administration during 3 months of pregnancy ☐ Oral administration 3 months before pregnancy to the first 3 months after pregnancy	brand______ duration（month）______<br>☐Once a day ☐2 times/day ☐3 times/day<br>☐Once a week ☐>3 times / week<br>Dosage per time________
Formula for pregnant women	☐No	☐ Oral administration 3 months before pregnancy ☐Oral administration during 3 months of pregnancy ☐ Oral administration 3 months before pregnancy to the first 3 months after pregnancy	brand______ duration（month）______<br>☐Once a day ☐2 times/day ☐3 times/day<br>☐Once a week ☐>3 times / week<br>Dosage per time________

Vitamin D supplements	☐No	☐ Oral administration 3 months before pregnancy ☐Oral administration during 3 months of pregnancy ☐ Oral administration 3 months before pregnancy to the first 3 months after pregnancy	brand______ duration（month）______ ☐Once a day ☐2 times/day ☐3 times/day ☐Once a week ☐>3 times / week Dosage per time________
DHA	☐No	☐ Oral administration 3 months before pregnancy ☐Oral administration during 3 months of pregnancy ☐ Oral administration 3 months before pregnancy to the first 3 months after pregnancy	brand______ duration（month）______ ☐Once a day ☐2 times/day ☐3 times/day ☐Once a week ☐>3 times / week Dosage per time________
Calcium milk powder	☐No	☐ Oral administration 3 months before pregnancy ☐Oral administration during 3 months of pregnancy ☐ Oral administration 3 months before pregnancy to the first 3 months after pregnancy	brand______ duration（month）______ ☐Once a day ☐2 times/day ☐3 times/day ☐Once a week ☐>3 times / week Dosage per time________

Part five Physical activity

Part one: day-to-day work

1. Are you currently working?

☐ Yes

☐ No(Skip to part 2: daily traffic)

2. In the past 7 days, how many days have you participated in moderate physical activities (such as lifting small items, cleaning, etc.) at work for more than 10 days minute? (Activities outside of work are not included)

________days / week

3. How long do you spend on moderate physical activity every day at work?

________ hours / day

________ minutes / day

4. In the past 7 days, how many days did you walk for more than 10 minutes? (note that walking time on the way to and from work is not included)

________ days / week

☐ No work-related moderate physical activity (Skip to part 2: daily traffic)

5. How long does it take to walk every day at work?

________ hours / day

________ minutes / day

Part two: daily traffic

6. In the past 7 days, how many days did you go out by car?

________ days / week

☐ Not going out by car (Skip to question 8)

7. How long does it take by car every day?

________ hours / day

________ minutes / day

8. In the past 7 days, how many days did you walk out for more than 10 minutes?

________ days / week

☐ Not walking out (Skip to part three)

9. How long did you it walk every day?

________ hours / day

________ minutes / day

Part three: daily life

10. In the past 7 days, how many days have you participated in heavy physical housework activities (such as carrying heavy objects, sweeping the floor, etc.) for more than 10 minutes? (excluding activities outside work)

________ days / week

☐ No work-related heavy physical activity (Skip to question 12)

11. How long do you spend on heavy housework every day?

________ hours / day

________ minutes / day

12. In the past 7 days, how many days have you participated in moderate physical housework activities (sweeping the floor, cleaning windows, etc.) for more than 10 minutes? (excluding activities outside work)

________ days / week

☐ No have moderate physical activity after work (Skip to the part four)

13. How long do you spend on moderate physical housework every day?

________ hours / day

________ minutes / day

Part four：Sports and recreation

14. In the past 7 days, how many days did you go out for a walk lasting more than 10 minutes? (The walking time described is not included)

________ days / week

☐ No going out for a walk (Skip to question 16)

15. How much time do you spend walking every day?

________ hours / day

________ minutes / day

16. How much time do you spend on bask in the sun every day?

________ hours / day

________ minutes / day

Part five: Sitting time

17. How much time did you spend sitting in your workday in the past 7 days?

________ hours / day

________ minutes / day

18. How much time did you spend sitting every day on weekends or rest days in the past 7 days?

________ hours / day

________ minutes / day

Part six: physical examination

**1**	**height**：□□□.□cm **weight**：□□□.□Kg **Pre-pregnancy weight:** □□□.□Kg<br>**waist circumference (cm)**： 1.□□□.□ 2. □□□.□ 3. □□□.□<br>**hip circumference (cm)**: 1.□□□.□ 2. □□□.□ 3. □□□.□
**2**	**hospital sphygmomanometer to measure blood pressure**<br>**(systolic blood pressure / diastolic blood pressure / heart rate)**:1. □□□ / □□□/ □□□

	**project team sphygmomanometer to measure blood pressure**: 1.□□□ / □□□/ □□□<br>**project team sphygmomanometer to measure blood pressure**: 2.□□□ / □□□/ □□□<br>**project team sphygmomanometer to measure blood pressure**: 3.□□□ / □□□/ □□□
**3**	**abdominal subcutaneous fat thickness：** □□□.□ mm **visceral fat thickness：** □□□.□ mm<br>**Signature of doctor：** ________
